# Supplementary material for: The Effects of Time-Restricted Eating versus Standard Dietary Advice on Weight, Metabolic Health and the Consumption of Processed Food: A Pragmatic Randomised Controlled Trial in Community-Based Adults
Source: Nutrients. 2021 Mar 23;13(3):1042. doi: 10.3390/nu13031042 (PMC8004978; doi:10.3390/nu13031042)
Supplement: Supplementary file 1 [file nutrients-13-01042-s001.pdf]

Supplementary Material

The effects of time-restricted eating vs. standard dietary advice on weight, metabolic health and the consumption of processed food: A pragmatic randomised controlled trial in community-based adults

Authors: Nicholas Edward Phillips[1], Julie Mareschal[2], Nathalie Schwab[3,4,5], Emily N.C. Manoogian[6], Sylvie Borloz[7], Giada Ostinelli[3,8,9], Aude Gauthier-Jaques[3], Sylvie Umwali[3,10], Elena Gonzalez Rodriguez[3,11], Daniel Aeberli[12], Didier Hans[11], Satchidananda Panda[6], Nicolas Rodondi[4,5], Felix Naef[1] and Tinh-Hai Collet[2,3,]*.

Affiliations
[1] Institute of Bioengineering, School of Life Sciences, Ecole Polytechnique Fédérale de Lausanne (EPFL), 1015 Lausanne, Switzerland.
[2] Service of Endocrinology, Diabetes, Nutrition and Therapeutic Education, Department of Medicine, Geneva University Hospitals (HUG), 1211 Geneva 14, Switzerland.
[3] Service of Endocrinology, Diabetes and Metabolism, Department of Medicine, Lausanne University Hospital (CHUV) and University of Lausanne, 1011 Lausanne, Switzerland.
[4] Department of General Internal Medicine, Bern University Hospital, Inselspital, University of Bern, 3010 Bern, Switzerland.
[5] Institute of Primary Health Care (BIHAM), University of Bern, 3012 Bern, Switzerland.
[6] Salk Institute for Biological Sciences, La Jolla, CA 92037, USA.
[7] Service of Paediatrics, Department Woman-Mother-Child, Lausanne University Hospital (CHUV) and University of Lausanne, 1011 Lausanne, Switzerland.
[8] Quebec Heart and Lung Institute Research Center (Centre de recherche de l'Institut universitaire de pneumologie et cardiologie de Québec), Québec, QC G1V 4G5, Canada.
[9] School of Nutrition (École de Nutrition), Laval University, Québec, QC G1V 0A6, Canada.
[10] Service of Obstetrics, Department Woman-Mother-Child, Lausanne University Hospital (CHUV) and University of Lausanne, 1011 Lausanne, Switzerland.
[11] Interdisciplinary Center for Bone Diseases, Service of Rheumatology, Lausanne University Hospital (CHUV) and University of Lausanne, 1011 Lausanne, Switzerland.
[12] Department of Rheumatology and Immunology, Bern University Hospital, Inselspital, and University of Bern, 3010 Bern, Switzerland.

* Corresponding author: Tinh-Hai Collet, MD, Service of Endocrinology, Diabetology, Nutrition and Therapeutic Education, Geneva University Hospitals (HUG), Rue Gabrielle-Perret-Gentil 4, 1211 Geneva 14, Switzerland. Tinh-Hai.Collet@hcuge.ch.

Table S1. Limited overlap of different food and drink categories

Categories	NOVA1 (%)	NOVA2 (%)	NOVA3 (%)	NOVA4 (%)	NOVA-A (%)	NOVA-C (%)	NOVA-S (%)	NOVA-D (%)
NOVA1	100	0.1	0.4	0.2	0	28	0	7.6
NOVA2	0.3	100	0.7	0.1	0	7.7	6.9	0
NOVA3	1.0	0.5	100	0.3	19	0	0.3	0.4
NOVA4	0.4	0.1	0.2	100	1.6	6.9	9.7	1.7
NOVA-A	0	0	90	9.7	100	0	6.8	0
NOVA-C	83	7	0	11	0	100	9	0
NOVA-S	0.2	28	1.9	70	8.1	40	100	0
NOVA-D	87	0.1	2.1	10	0	0	0	100

Footnotes: For each row, all ingestion events are filtered according to the category in the row name. Each column value represents the percentage of other categories that are present after filtering on the row name. Categories follow and extend the NOVA classification: NOVA1: "Unprocessed or minimally processed foods," NOVA2: "Processed culinary ingredients," NOVA3: "Processed foods," NOVA4: "Ultra-processed foods," NOVA-A: "Alcohol-containing drinks," NOVA-C: "Caffeinated drinks," NOVA-S: "Sweet drinks," NOVA-D: "Other drinks."

Table S2. Effects of TRE and SDA on eating behaviour

	Time-restricted eating (TRE)			Standard dietary advice (SDA)			Between-group difference (95% confidence interval) [c]	P for comparison of pre-post changes between TRE and SDA [b]
**Mean (SD)** [a]	**Pre-intervention**	**Post-intervention**	**P for comparison (pre-post)** [b]	**Pre-intervention**	**Post-intervention**	**P for comparison (pre-post)** [b]		
NOVA1, %	43.5 (13.0)	41.9 (13.9)	0.35	37.8 (12.5)	44.8 (13.2)	< 0.01	-8.6 (-14.1; -3.2)	< 0.01
NOVA2, %	14.7 (6.0)	15.8 (6.4)	0.37	12.4 (5.8)	13.9 (5.3)	0.09	-0.5 (-3.3; 2.4)	0.76
NOVA3, %	17.5 (7.9)	19.3 (8.7)	0.10	18.0 (7.1)	17.1 (5.4)	0.40	2.7 (-0.2; 5.6)	0.08
NOVA4, %	24.2 (11.1)	23.0 (9.0)	0.48	31.9 (11.4)	24.2 (11.8)	< 0.01	6.4 (0.4; 12.4)	0.04
NOVA-A, %	4.4 (5.3)	3.9 (4.8)	0.17	4.4 (4.5)	3.2 (3.4)	< 0.01	0.6 (-0.5; 1.7)	0.27
NOVA-C, %	19.6 (11.4)	17.9 (11.7)	0.28	17.4 (6.8)	13.9 (8.7)	0.07	1.8 (-2.8; 6.5)	0.44
NOVA-S, %	4.5 (7.3)	3.0 (4.6)	0.08	4.4 (4.7)	4.6 (6.8)	0.74	-1.8 (-3.9; 0.3)	0.12
NOVA-D, %	3.9 (4.3)	2.2 (2.5)	0.02	3.9 (3.2)	3.8 (3.5)	0.85	-1.6 (-3.2; -0.0)	0.06
Eating duration, hours [d]	15.48 (1.14)	12.50 (1.29)	< 0.01	15.17 (0.96)	14.89 (1.21)	0.16	-2.70 (-3.47; -1.93)	< 0.01
Eating start, hours [d]	6.83 (0.67)	8.36 (1.00)	< 0.01	6.96 (0.99)	6.86 (1.08)	0.40	1.63 (1.12; 2.13)	< 0.01
Eating midpoint, hours [d]	13.84 (1.44)	13.64 (1.31)	0.59	13.88 (1.18)	13.05 (0.88)	< 0.01	0.62 (-0.24; 1.47)	0.18
Eating end, hours [d]	22.31 (1.17)	20.86 (1.03)	< 0.01	22.13 (0.95)	21.76 (1.10)	0.01	-1.07 (-1.62, -0.53)	< 0.01

Footnotes: [a] Categories follow and extend the NOVA classification: NOVA1: "Unprocessed or minimally processed foods," NOVA2: "Processed culinary ingredients," NOVA3: "Processed foods," NOVA4: "Ultra-processed foods," NOVA-A: "Alcohol-containing drinks", NOVA-C: "Caffeinated drinks," NOVA-S: "Sweet drinks," NOVA-D: "Other drinks." [b] All p for comparisons were calculated with the Student t-test. [c] Mean difference comparing pre-post change between the TRE and SDA groups (n = 25 and 20, respectively). [d] Eating duration, the start of the eating interval, the median of the eating interval, and the end of the eating interval, presented as fractional hours, i.e. 1.33h = 1 hour and 20 minutes.

Table S3. Baseline characteristics by randomisation group

	**SDA (n = 26)**	**TRE (n = 28)**	**Both arms (n = 54)**
**Demographics**			
Age, mean (SD)	42.5 (14.0)	44.3 (12.8)	43.4 (13.3)
Marital status [a], n (%)			
Married, partnership	17 (68%)	23 (92%)	40 (80%)
Single, separated, widow-er	8 (32%)	2 (8%)	10 (20%)
Education, n (%)			
Primary, secondary	4 (15%)	2 (7%)	6 (11%)
Apprenticeship, vocational	5 (19%)	7 (25%)	12 (22%)
Professional school	2 (8%)	5 (18%)	7 (13%)
University, or higher degree	15 (58%)	14 (50%)	29 (54%)
Born in Switzerland, n (%)	21 (81%)	19 (68%)	40 (74%)
**Clinical characteristics**			
Weight, kg, median (IQR)	75.2 (67.9–83.6)	79.3 (68.5–85.2)	77.5 (68.0–84.7)
BMI, kg/m^2, median (IQR)	27.7 (23.8–30.3)	28.3 (25.2–31.6)	28.3 (24.6–30.5)
BMI < 25.0 kg/m^2, n (%)	8 (30%)	6 (21%)	14 (26%)
BMI 25.0–29.9 kg/m^2, n (%)	9 (35%)	13 (46%)	22 (41%)
BMI ≥ 30.0 kg/m^2, n (%)	9 (35%)	9 (32%)	18 (33%)
WC, cm, median (IQR)	87.5 (81.0–100.0)	92.0 (86.0–99.0)	90.3 (84.0–100.0)
Current smokers, n (%)	2 (8%)	3 (11%)	5 (9%)

Cardiovascular disease [b], n (%)	1 (4%)	2 (7%)	3 (6%)
Endocrine disorder [c], n (%)	1 (4%)	2 (7%)	3 (6%)
Respiratory disorder, n (%)	4 (15%)	5 (18%)	9 (17%)
**Metabolic syndrome (MS)** [d]			
MS components, n (%)			
1. Central obesity	20 (77%)	23 (82%)	43 (80%)
2. Hypertension	12 (46%)	11 (39%)	23 (43%)
3. Impaired fasting glucose	6 (23%)	6 (21%)	12 (22%)
4. High triglycerides	6 (23%)	6 (21%)	12 (22%)
5. Low HDL-cholesterol	7 (27%)	8 (29%)	15 (28%)
MS by the IDF definition, n (%)	6 (23%)	7 (25%)	13 (24%)
**Lifestyle**			
Sleep duration [e], hours, mean (SD)	7.15 (1.01)	7.23 (0.93)	7.19 (0.96)
Sleep quality [f], mean (SD)	5.1 (2.4)	5.4 (2.8)	5.2 (2.6)
Physical activity [g], median (IQR)	1053 (678–2226)	1346 (570–1907)	1191 (594–1920)

Footnotes: Related to Table 1. [a] The data on marital status were available for 50 individuals. [b] Cardiovascular disease prior to the inclusion in the study, e.g. coronary heart disease, cerebrovascular disease, peripheral artery disease. [c] Mostly hypothyroidism with thyroxine replacement at a stable dosage. [d] The metabolic syndrome (MS) and its components were defined as follows: Central obesity was defined as body mass index (BMI) ≥ 30 kg/m^2 or waist circumference (WC) ≥ 80 cm (women) or WC ≥ 94 cm (men); hypertension as systolic blood pressure (BP) ≥ 130 mmHg and/or diastolic BP ≥ 85 mmHg; impaired fasting glucose for plasma levels ≥ 5.6 mmol/L (100 mg/dL); high triglycerides if ≥ 1.7 mmol/L (150 mg/dL); and low HDL cholesterol if < 1.29 mmol/L (50 mg/dL, women) or < 1.03 mmol/L (40 mg/dL, men). [e] Weighted average of sleep duration between work days and off days. [f] Sleep quality was assessed with the Pittsburgh Sleep Quality Index (PSQI), ranging from 0 (no issues) to 21 points (extreme disturbance of sleep quality). [g] Physical activity was assessed among 51 individuals with the International Physical Activity Questionnaire (IPAQ), expressed as MET-minutes per week.

Figure S1. Study design

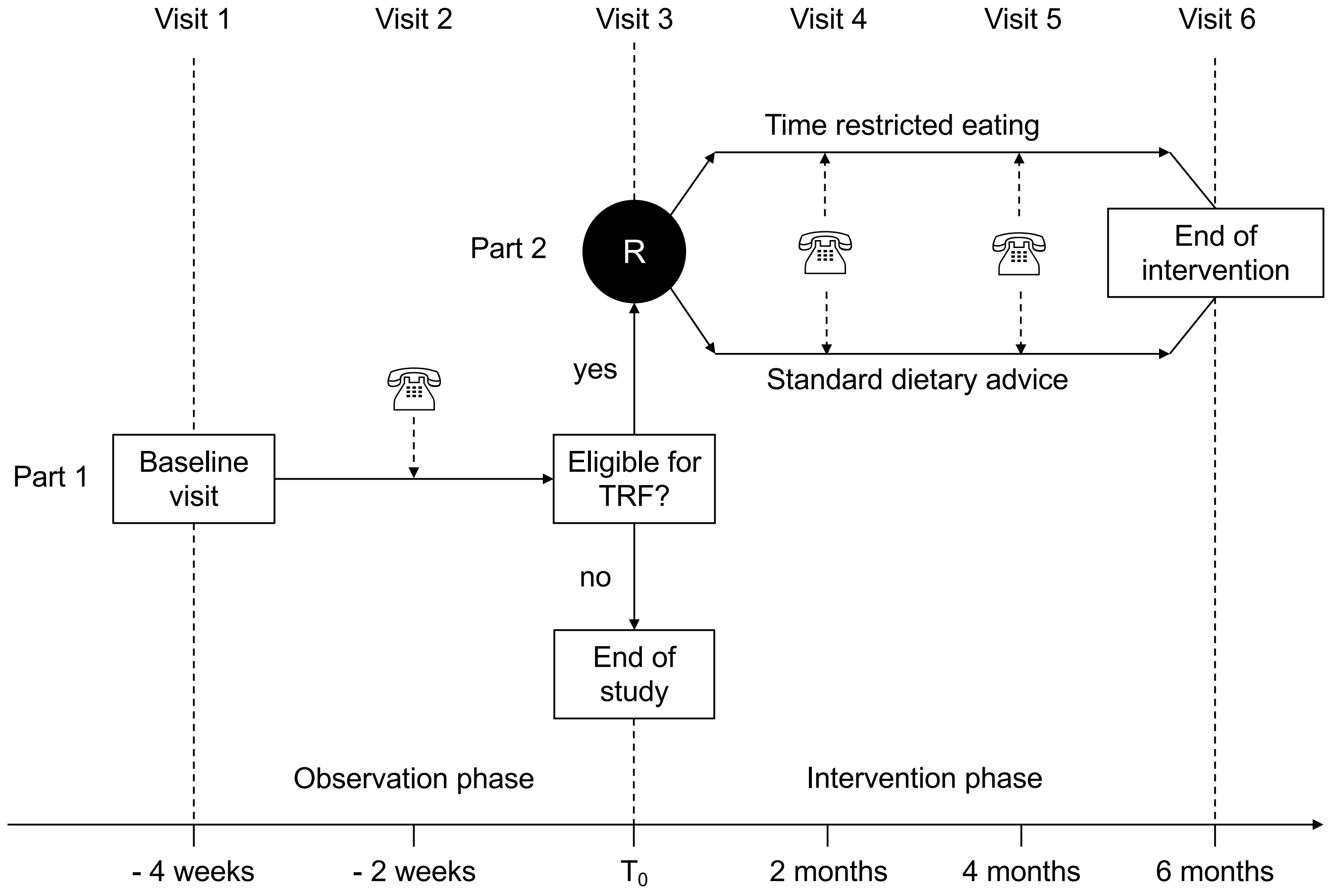

<u>Legend</u>: Related to the Methods, section 2.1. Study design and flow of participants through the 4-week observation phase, followed by a 6-month intervention phase if they were eligible (see text). In addition to the 3 encounters in person (visits 1, 3, and 6), interim contacts were made over the phone or via email to ensure compliance to the study protocol (visit 2) and record weight measured at home (visits 4 and 5).

Figure S2. The time profile of ingestion events

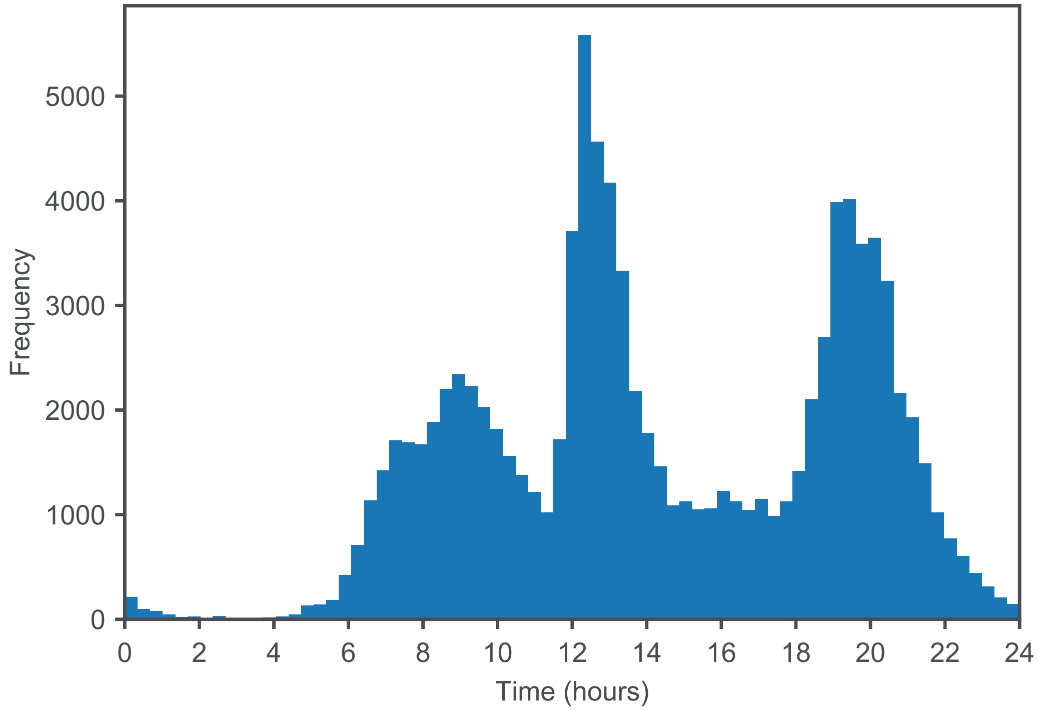

<u>Legend</u>: Related to the Methods, section 2.2. The number of recorded ingestion events across all participants as a function of clock time.

Figure S3. Changes in secondary clinical outcomes after TRE and SDA interventions

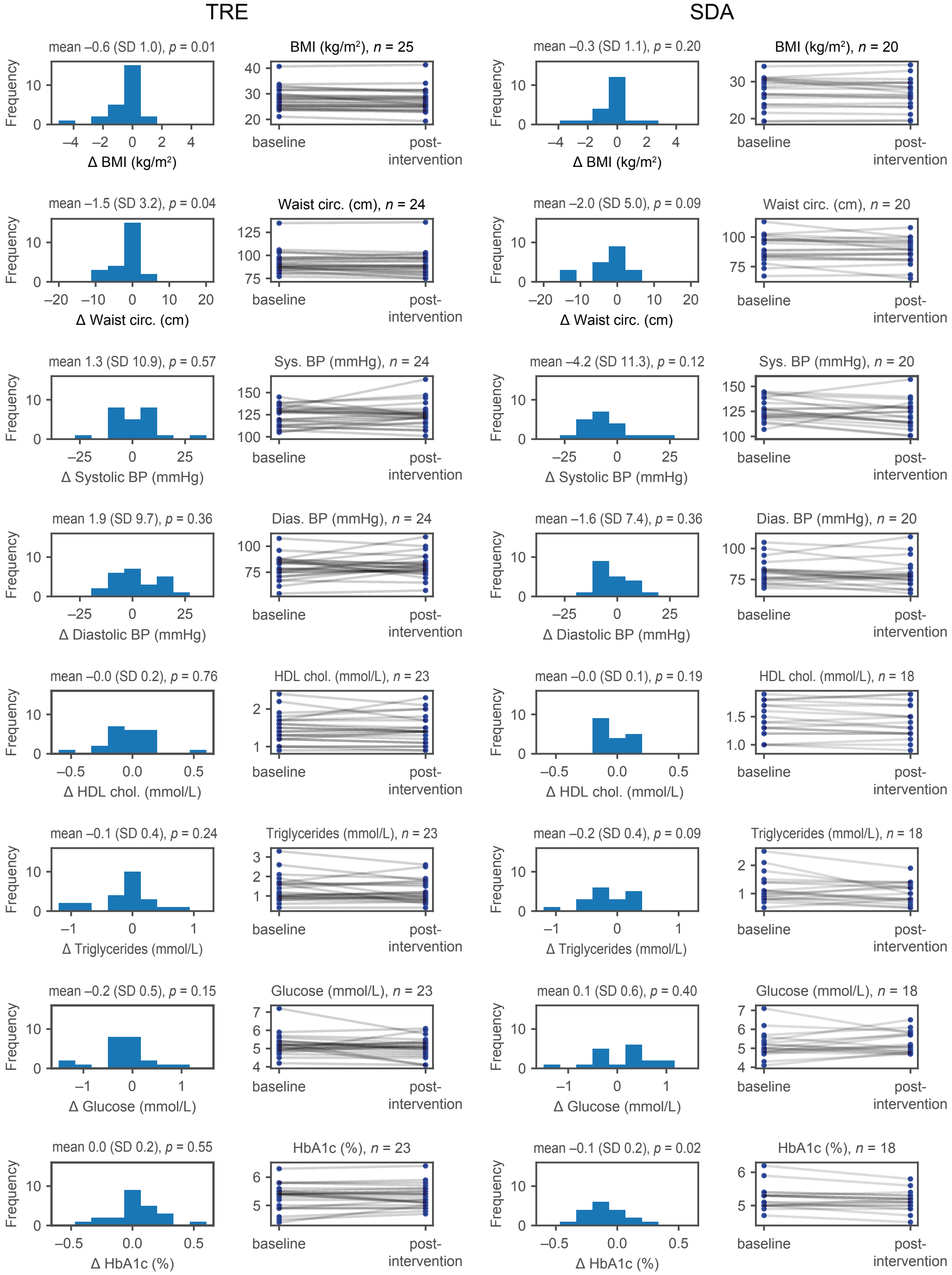

<u>Legend</u>: Related to Table 2 & Figure 4. Changes in body mass index (BMI), waist circumference, systolic and diastolic blood pressure (BP), HDL cholesterol, triglycerides, fasting plasma glucose and glycated haemoglobin (HbA1c) after time-restricted eating (TRE, left columns) and standard dietary advice (SDA, right columns). Data are presented as histograms (changes in % from baseline) and individual trajectories pre-/post-intervention. Numbers above each histogram represent the changes (unit on the x-axis) as mean, standard deviation, and p-value.

Figure S4. Changes in body composition after TRE and SDA interventions

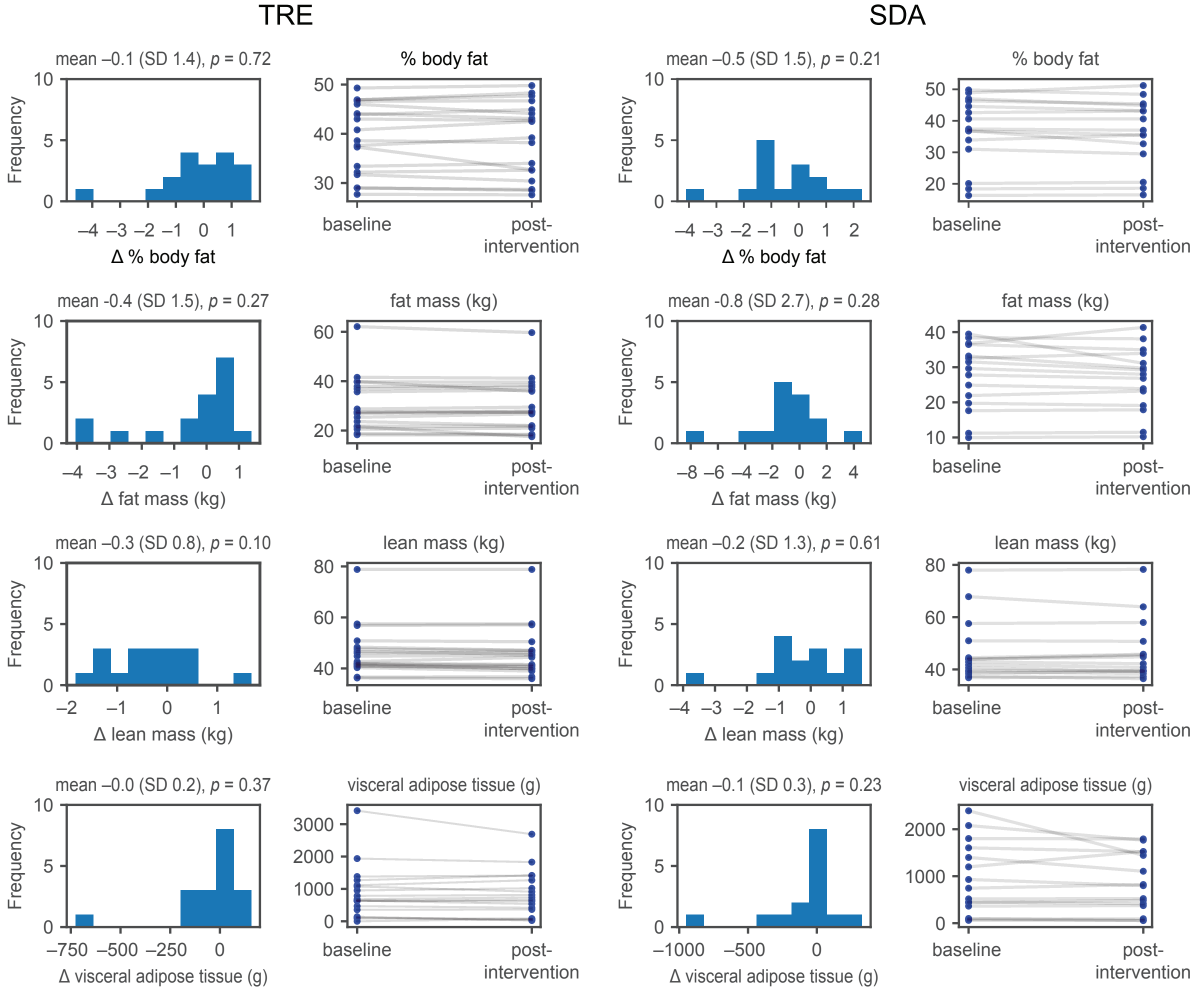

Legend: Related to Figure 4. Changes in body composition by dual energy X-ray absorptiometry, expressed as the body fat percentage (in % points), fat mass (in kg), lean mass (in kg), and visceral adipose tissue (in grams), after time-restricted eating (TRE, left columns, n = 18) and standard dietary advice (SDA, right columns, n = 15). Data are presented as histograms (changes in % from baseline) and individual trajectories pre-/post-intervention. Numbers above each histogram represent the changes (unit on the x-axis) as mean, standard deviation, and p-value.
